# Toward Quantifying the Psychopathology of Eating Disorders From the Autonomic Nervous System Perspective: A Methodological Approach

**DOI:** 10.3389/fnins.2019.00606

**Published:** 2019-07-01

**Authors:** Neide Simões-Capela, Giuseppina Schiavone, Walter De Raedt, Elske Vrieze, Chris Van Hoof

**Affiliations:** ^1^ESAT, KU Leuven, Heverlee, Belgium; ^2^IMEC, Heverlee, Belgium; ^3^IMEC-NL, Eindhoven, Netherlands; ^4^University Psychiatric Center, KU Leuven, Leuven, Belgium

**Keywords:** eating disorders, autonomic nervous system, psychometrics, ecological momentary assessment, wearables, stress

## Abstract

The phenomenology of Eating Disorders (ED) relates with altered functioning of the Autonomic Nervous System (ANS). The lack of agreement in what comes to the direction and significance of such alterations is possibly due to the variability in the ED spectrum. As the stress response system is an integral part of the ANS, we propose to investigate ANS tonic variations and phasic activations in response to stressors. We hypothesize that, while using stress as a test probe, characteristic ANS dysregulations in ED may be found when considering several physiological signals measured over time, and weighted by the individual psychological profiles. In this article we describe a novel methodological approach to investigate this hypothesis with the aim of providing further clarification on the ED spectrum conceptualization. The proposed methodology has been designed to be easily integrated in clinical practice and, eventually, in daily life. The population under observation includes both patients in treatment for ED, and matched controls. The study session has the duration of 1 day, including: (1) the administration of a stress task in a controlled environment and (2) naturalistic data collection. The stress task is designed to elicit both mentally and physically driven ANS activation. The naturalistic component intends to illustrate the psychophysiology in everyday life. We use wearable devices to continuously and non-invasively measure bio-signals related to ANS functioning. This information is complemented with psychometric information from validated stress and ED scales and ecological momentary assessments. The protocol has received ethical approval and has been implemented in practice, currently accounting for 37 patients (out of 120) and 16 controls (out of 60). Ongoing work focus on the definition and implementation of a data processing pipeline to quantitatively test our hypothesis, both standard statistical methods and more exploratory machine learning approaches will be considered.

## Introduction

Anorexia Nervosa (AN) and Bulimia Nervosa (BN) constitute the official ED diagnoses according to the Diagnostic and Statistical Manual of Mental Disorders (DSM-IV)^1^ (American Psychiatric Association, 2000). Partial AN/BN syndromes and the prominent Binge Eating Disorder (BED) are included in a residual and highly heterogeneous subset of ED, the Eating Disorders Not Otherwise Specified (EDNOS). ED markedly influence the physical and mental well-being of sufferers. AN is associated with intense fear of fatness, distorted body image, and exaggerated dieting, leading to severe weight loss. BN features recurrent food binges followed by compensatory behaviors (e.g., purges, excessive exercise, or fasting) to avoid weight gain. BED regards frequent episodes of fast intake of exaggerated food portions, accompanied by feelings of lack of control (American Psychiatric Association, 2013a). While not well-documented by epidemiological studies, it is systematically reported that most ED cases fall in the EDNOS group (Machado et al., 2012; Smink et al., 2012; Vo et al., 2017). We know that only a minority of ED cases reaches the health care system (Keski-Rahkonen and Mustelin, 2016), even though most sufferers receive treatment related to unspecific emotional problems (Hudson et al., 2007). ED etiology is multifactorial with more than 70% of patients having psychiatric comorbidities and often switching across diagnosis within the ED spectrum over time (Hudson et al., 2007; Larranaga et al., 2012; Keski-Rahkonen and Mustelin, 2016). Remission occurs after at least 4 years (Keel and Brown, 2010; Kessler et al., 2013). Therefore, in order to comprehensively improve treatment outcome, we may need to better grasp the seemingly dimensional underlying psychopathological mechanisms of these illnesses.

The official guidelines for classification of psychiatric pathologies are described in the DSM (American Psychiatric Association, 2000, 2013b). Disorders are presented as categories and symptoms are evaluated in a syndromic manner. However, while it has been useful as a basis for communication among clinicians and researchers, the DSM validity has long been discussed, and after several updates it is still flawed (Vo et al., 2017). In general, the categorical conceptualization of mental disorders does not rely on a grounded neuro-psychopathological basis, and has been reduced to a matter of opinion or preference (Haslam, 2003; Williamson et al., 2005). Several studies have explored a dimensional perspective, i.e., the idea of a continuous spectrum, to model mental health disorders (Haslam, 2003) and in particular ED (Williamson et al., 2005). Explorations were grounded solely on information from self-reports and psychometric data (no physiological data). According to preliminary results, both categorical and dimensional perspectives may need to be combined in order to describe the mental health disorders spectrum (Williamson et al., 2005).

Arousal and regulatory systems are among the five Research Domain Criteria (RDoC) appointed as potentially valid dimensional constructs underlying psychopathology (Cuthbert, 2014). The arousal system facilitates interactions with the environment in a context-specific manner and has associations to physical fitness (Huang et al., 2013). In psychophysiology, arousal is defined as the response to a perturbation (i.e., a stressor) that may create a homeostatic deregulation of the organism (Koolhaas et al., 2011). While acute arousal can be accommodated by physiological processes, prolonged activation can result in pathological states.

In the human body, specific neuroendocrine paths are triggered causing arousal (Kemeny, 2003; Lo Sauro et al., 2008). The first one involves the ANS (Kemeny, 2003), a part of the Peripheral Nervous System concerning involuntary bodily processes. The ANS encompasses two branches working in opposition to regulate the body equilibrium: the sympathetic branch associated with arousal and the flight-or-fight (FoF) response, and the parasympathetic associated with rest-and-digest states (McCorry, 2007). The FoF is a full body effort to increase blood flow to skeletal muscle, causing considerable effects at a physiological level. It leads to increased heart rate (HR); vasoconstriction in the gut and kidneys to divert blood to the muscles; bronchodilation to facilitate oxygen uptake and carbon dioxide outtake; increase of glucose and fatty acid concentrations in the blood to leverage metabolic needs; generalized sweating for thermoregulation; pupil dilation to allow more light in and adapt vision to focus in the distance. Considering such phenomena, ANS arousal can be described on the basis of pupillary dilation, muscle activity, cardiac activity, respiration rate, blood pressure (BP), skin conductance (SC), and skin temperature (Kemeny, 2003; McCorry, 2007).

Physiological substrates involved in ANS regulation are altered in ED (Westmoreland et al., 2016), for instance decreased HR positively related to lower metabolic rate (Buchhorn, 2016), HRV is inversely related to body weight (Karason et al., 1999; Mazurak et al., 2011), lower skin conductivity relates to dehydration caused by reduced fluid intake or purge, decreased skin temperature is linked to poor circulation induced by reduced cardiac activity. ED onset is often preceded by a specific stressful event (Wheat et al., 2000; Cabras et al., 2008), and the disease maintenance has also been connected to chronical arousal states, linked to specific contexts triggering psychopathological behavior (Wheat et al., 2000).

Literature studies on ANS function in ED report two main study conditions: (1) baseline observations, e.g., at rest or respecting to slow tonic variations; (2) task induced, e.g., in reaction to a stressor. In summary, most studies point out the existence of baseline ANS dysregulations, that are transversal to AN and BN (Mazurak et al., 2011; Buchhorn, 2016; Chudecka and Lubkowska, 2016; Peschel et al., 2016). These dysregulations in AN and fasting BN have been documented as a parasympathetic overactivation with a correspondent sympathetic withdrawal. Task induced responses, mostly show no differences across healthy and ED samples (AN, BN, BED, and EDNOS) (Vocks et al., 2007; Vögele et al., 2009; Hilbert et al., 2011), though, inconsistencies are reported (Koo-Loeb et al., 2000). Additionally, AN and BN are better documented than BED and EDNOS in what comes to ANS alterations. Cardiovascular parameters (HR and HRV) are the most well-explored descriptors of ANS function in ED, both at baseline and in reaction to stressors. Temperature, BP, and SC are obtained with short measurement protocols, so their use toward pervasive follow-up is not well-explored. Small sample size is indicated in most studies as a limitation, and short cross-sectional measurements also pose limitations toward fully understanding ANS functioning in ED. Overall these studies recommend that considerations should be given to age, body mass index (BMI), comorbidities, associated clinical complications (e.g., starvation, malnourishment, and low body weight), types of eating disordered behaviors (e.g., vomiting), illness duration (chronic or acute), phase of the disease being considered (Mazurak et al., 2011), disorder subtypes (Peschel et al., 2016), and medications.

Our goal is to investigate the tonic and phasic activation of the ANS across the ED spectrum, to discover associations between the psychopathology and arousal states, eventually recurring to advanced data fusion techniques. In this paper, we describe the methodological approach in the basis of our investigation. Answering questions related to whether ANS dysregulations reflect ED psychopathology, are a side effect of the medical complications of the disease (e.g., vomiting and starvation), or both, is out of our scope.

Our work aims at bridging some of the research gaps found in the literature, namely: (1) the full ED spectrum has only punctually been studied using the same psychophysiological protocol (Vocks et al., 2007); (2) physical fitness is usually not assessed in psychophysiological studies and is especially relevant in ED, as it can vary across extremes; (3) bio-signals from several sources have previously been measured in controlled tasks (Vocks et al., 2007; Vögele et al., 2009; Hilbert et al., 2011), though, conclusions were established based on each source separately, without considering data fusion strategies; (4) finally, there has been no report on the study of ED based on bio-signals collected in naturalistic settings. With the general mindset of providing tools to leverage the clinicians work, while testing possibilities that can enhance the follow-up in naturalistic conditions, we employ tools that could be easily integrated as a part of clinical practice. Ecological Momentary Assessment (EMA) to gather information on activity, food consumption, and feelings, if proved useful, can be integrated in a smartphone application. The wearables used for non-invasive autonomic sensing are the most compact and discreet non-invasive sensors in the market, as compared to sensing modalities, such as autonomic Electromyogram (EMG) or central Electroencephalogram (EEG).

We hypothesize that the ED state may be differentiated from a healthy state using a multiparameter approach to data analysis based on psychological and physiological signals. We also intend to test if ED psychopathology can be indexed based on such information. Both hypotheses will be tested on controlled and ambulant data. The following guiding research questions were established to decomposed and test our hypotheses:

- Do ED patients present characteristic autonomic activation during the laboratory stress task, when compared to healthy controls?
- Do autonomic signals during reported high stress moments, in an ambulant condition, reflect the laboratory analysis results (i.e., do both groups still differ according to the previously identified, parameters if any)?
- Can the four most prominent ED classes (i.e., AN, BN, BED, and EDNOS) be identified based on autonomic signals?
- Based on a data driven approach, relying both on psychological descriptors and ANS signals, can we establish a multiparameter model of the ED spectrum (i.e., AN, BN, BED, and EDNOS) that describes psychopathological state?

In the following sections we will present the experimental design (including study population, protocol description, power calculation) (section Experimental Design), discuss the limitations of our approach and suggest possibilities for further development (section Discussion).

## Experimental Design

Toward studying the ANS in ED we devised an experiment that would allow us to capture both tonic and phasic ANS activation using the following conditions: (1) at rest in the lab; (2) in response to a stressor in the lab; and (3) in naturalistic conditions, where both rest and stress situations may occur. During the day of the study participants are administered a stress task in a controlled environment, and afterwards they continue freely with their routines for the rest of the day. Measurements of autonomic physiology using wearable sensors are pervasive throughout the day, and EMA on stress, feelings and activities take place every hour.

The detailed methodology is described in the next sections. The protocol was approved by the Medical Ethical Committee of U. Z. Leuven (Belgium), and the study has been running ever since February 2018, with an amendment accepted on January 2019 toward extending the number of centers where subjects are recruited.

### Study Sample

The study sample is composed of a group of ED patients (clinical group) and a group of healthy subjects (control group). Detailed admission criteria for each group are displayed in Table 1.

**Table 1:** Admission criteria: inclusion and exclusion criteria applied for the selection of the participants on the study.

	**Study group**	**Control group**
Inclusion criteria	Be a female; Having current ED complaints; Being currently treated for ED; Have in between 18 and 50 years old;	Be full-time employee in a company, doing office work; Match in gender, age and BMI one subject in the study sample;
	Have the ability and will to report on feelings several times during the day.
Exclusion criteria	Suffer from major chronical medical condition requiring continued medication other than that used in the treatment of the ED and its comorbidities;	Be a night shift worker; Have history (past or present) of diagnosed mental health conditions or complaints; Have major stress complaints; Suffer from major chronic medical condition that requires continued medication;
	Suffer from acute medical conditions; Suffer from dyslexia or dyscalculia; Be pregnant; Carry any implanted devices; Have conditioned mobility; Be under the effect of medication that impairs the mental state or lack the intellectual capabilities to an extent that prevent the provision of an informed consent to participate.

The clinical group includes patients (inpatient, day hospital/program, or ambulant regimen) receiving treatment for their ED at a medical unit in Belgium. In the treatment context, all patients, including the ones admitted at the hospital, have free time for rest or social interactions, and can leave the treatment facilities. Mealtimes are fixed in the inpatient and day hospital situations. To pinpoint characteristic features of ED only subjects in treatment are considered, when there is irrefutable need for clinical attention. To gather information on the psychopathology across the ED spectrum, we include patients with strict diagnoses (i.e., AN, BN, BED), but also those falling within EDNOS. These classifications are primarily provided by the clinician. Since we intend to focus on characteristic ED traits and its comorbidities, we exclude subjects with coexistent health issues not directly connected to the core features of ED, such as somatic chronical illness with current impact in daily life or need for continued medication, and acute illness. Knowing from epidemiological studies that women are the major group in ED (Larranaga et al., 2012; Smink et al., 2012), and having empirical evidence that men in treatment for ED are relatively sparse, we opted to include only females to establish a grounded statistical analysis. Age was limited to a period that should be stable in terms of major or definitive endocrine changes. We exclude pregnant women related to their special endocrine state. We exclude carriers of implanted devices (e.g., pacemaker) to avoid interference with the wearables. We exclude individuals with dyslexia, dyscalculia, and conditioned mobility, which could interfere with, the execution of the Stroop-Color Word, the arithmetic and the fitness tasks, respectively.

The control group is composed of full-time employees in a 9 to 5 desk job, a condition that will allow us to compare the period of activity and the activity intensity with that of the patients in the hospital. Individuals in this group cannot display major health issues that impact their daily life or that require continued medication, nor should they have history of mental health issues.

**Figure 1:**
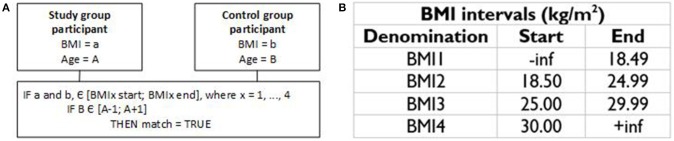
BMI and age matching schemes: **(A)** pseudo-code; **(B)** BMI ranges according to WHO.

Controls are matched to patients according to gender, age and BMI ranges, in accordance to the scheme in Figure 1. The BMI intervals proposed by the World Health Organization (WHO) (World Health Organization, 2018) were considered to establish approximate BMI matches, as exact matches would be unpractical for the extremes found in AN and BED that cannot find parallel in the healthy population. For age matching we consider an interval of ±1 year around the age of the patient, toward maintaining identical demographics in both groups, under the assumption that the difference of 1 year is not biologically relevant in the range considered. With this scheme we intend to maintain comparable anthropometrics across groups.

### Protocol

The study is conducted over two separate days of contact (see Table 2): the recruitment day and the study day. In the first day of contact, the participant indicates the language preference (materials are available in English and Dutch), provides legal consent for data to be acquired and is screened toward verifying the admission criteria. A second day of contact is only scheduled if the participant abides to the admission criteria. The procedures related to the study day take place in the period from 9 a.m. to 5 p.m. The first 1 h 30 min takes place in a controlled environment, during which the study procedures are introduced, devices are setup, the participant is asked to fill in a set of standardized psychometric questionnaires and is administered a multimodal stress task. For the next 6 h 30 min, the participant continues a regular daily routine in ambulant environment. Measurements of autonomic physiology using wearable sensors are pervasive during this day and EMA on stress, feelings and activities takes place at each hour. In the clinical group weight is assessed during this day. In the control group, the weight is evaluated on the first visit to allow the BMI match to be found, and then assumed stable across sessions. The clinician (or the patient) provides a clinical report on the symptomatology being experienced and the type of ED being managed. Both groups abide to similar procedures, described in a standard operating procedure that guides the researchers and clinicians involved in the study.

**Table 2:** Study overview: recruitment day and study day.

**Recruitment day (1 h)**	**Study day (1 h 30 contact + 6 h 30 free living)**
Informed Consent Form Verification of admission criteria Structured diagnostic interview	9:00: introduction to study procedures, sensor setup 9:15: standardized questionnaires, 1st diary (EMA) report ~9:45: stress task ~10:30: ambulant 17:00: diary and sensors are collected, participants are debriefed, remuneration is delivered, clinical information is retrieved.

#### Sensing Devices

**Figure 2:**
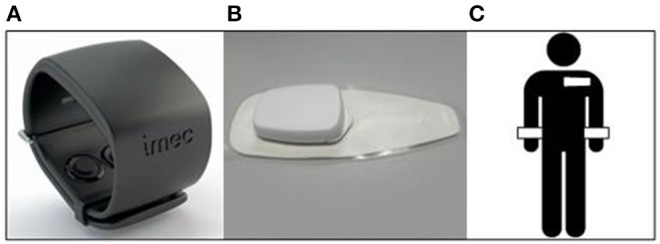
Wearable sensors: **(A)** Chillband, **(B)** ECG patch, and **(C)** sensor positioning.

Two wearable sensors are employed in this study: (1) the Chillband (IMEC vzw, Belgium) is a wrist worn sensing device that captures SC, skin temperature and acceleration (Figure 2A), at sampling frequencies of 256, 1, and 32 Hz, respectively; (2) the Electrocardiogram (ECG) patch (Biotelemetry, Denmark) is a sensing node attached to an adhesive chest patch, it captures ECG and acceleration (Figure 2B) at a sampling frequency of 256 and 32 Hz, respectively. Both wearables were previously employed in a large-scale study on daily stress in office workers (Boucsein et al., 2012). These wearables were the most compact and discreet non-invasive sensors in the market at the time of the trial setup. Despite being relevant and frequently used in laboratory settings, EMG and BP are less convenient for measurements in daily life and they were not considered for this study. During the study day participants carry three devices: two Chillbands and one ECG patch, placed according to the depiction in Figure 2C. The rational of employing two wrist sensors lays on the fact that wrist SC has been shown to diverge across both sides of the upper body (Picard et al., 2015), and this can provide important information on the arousal states in ambulant conditions.

#### Clinical Information

In the clinical group, the clinician responsible for the patient fills in a form with clinical information, including the treatment program (i.e., inpatient, day hospital/program, ambulant), days since admission, age, height, weight at start of treatment, weight on the day of the study session, diagnose according to clinical evaluation, symptomatology, and current medication. In the case of ambulant patients, if the clinician cannot be reached, the form is filled in by the researcher based on the patient report.

#### Standardized Psychometric Tools

Four standardized psychometric tools are employed in this study to model the participants' psychology: the MINI 5.0, a diagnostic interview; the EDI3 (diagnostic list and scale), a self-report to assess ED psychopathology; the DEBQ, a self-report to assess general eating style, and behaviors; and the PSS10, a self-report on stress. These tools have been highly validated and are widely used in trials. Table 3 describes each of them and their purpose in the context of this study.

**Table 3:** Description of standardized psychometric tools and their purpose in our study context.

**Tool**	**Description**	**Purpose**
Mini International Neuropsychiatric Interview (MINI)	Structured diagnostic interview to asses mental health disorders (Sheehan et al., 1998) according to DSM guidelines, with yes/no answers. Takes 15–60 min to complete, depending on the answers. Scores high in validation and reliability Lecrubier et al., 1997, and it has been widely used in research.	MINI-screen version 5.0 is employed to refute the existence of mental health disorders in the control group^a^, and to assess the ED typology and psychiatric comorbidities in the clinical group.
Eating Disorders Inventory (EDI3)	Fifteen minutes self-report focusing on psychological dimensions with clinical relevance for ED. Includes a diagnostic list appendix to assess demographics and anthropometrics. It is one of the most used tools in ED research Túry et al., 2010 and its psychometric properties are satisfactory Clausen et al., 2011.	EDI3 and the diagnostic list are delivered to both study groups to quantify the ED psychopathology.
Dutch Eating Behavior Questionnaire (DEBQ)	Ten minutes self-report. Includes three scales (emotional, restrained and external eating) relevant to characterize eating behaviors. Proven successful in assessing eating style traits that are relevant for ED assessment Wardle, 1987.	DEBQ is employed to gather a general description of eating behaviors, as EDI3 concepts are specific to ED and may be extraneous to non-ED subjects It is delivered to both study groups, but is specially relevance to describe controls, who may present behavioral nuances not captured in EDI3.
Perceived Stress Scale (PSS10)	Assesses individual stress levels during the past month. Five minutes self-report consisting of 10 questions, inquiring how often a certain feeling was experienced on a scale of 0 (never) to 4 (very often). Considered an effective indicator of the degree in which life events are perceived as stressful Kamarck et al., 1983.	Used toward quantifying stressful events that occurred prior to the study. This information is not covered by ED related assessments. It goes along with our view on the connection of stress, ANS dysregulations and ED.

a *If a control subject fulfils the symptoms for a MINI diagnosis then she is excluded, even after passing the initial assessment*.

#### Ecological Momentary Assessment

Ecological Momentary Assessment is a psychometric method intended at overcoming the limitations of retrospective self-reports, that rely on recollection of past events and may be biased. This is accomplished by regularly assessing the individuals' state with short questions, providing a close to real time assessment in a naturalist setting (Keel and Brown, 2010). In the past decade, EMA has been increasingly used to study ED, it has been shown useful in enhancing the empirical understanding on the disease and is regarded as powerful instrument to devise personalized and just-in-time interventions (American Psychiatric Association, 2013a).

**Figure 3:**
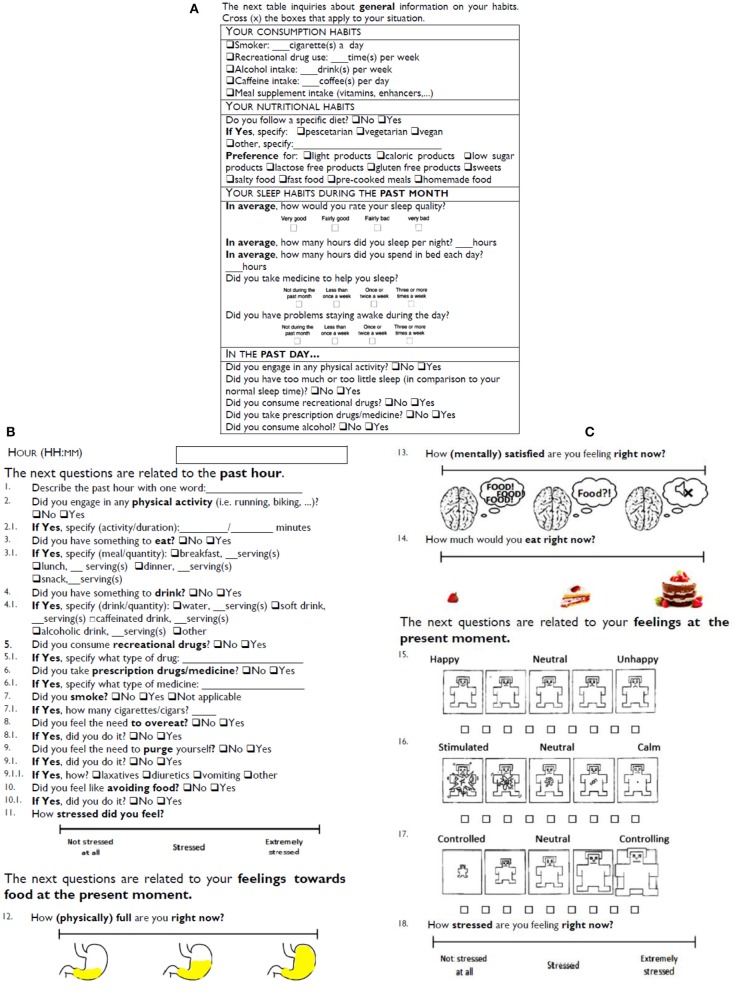
Diary: **(A)** Background, **(B,C)** Hourly report.

In this study the EMA consists in a paper diary (cf. Figure 3) that is filled in at every hour, from 9 h 30 to 16 h 30, totaling eight reports. As a reminder, an hourly alarm is set on the participant's phone. The diary includes a one-time report about consumption, nutrition and sleeping habit (Buysse et al., 1989), and assessment of events occurred during the past day that may affect the study results (e.g., abnormal sleep duration, alcohol consumption). The first encompasses: activities; food, drink, and medicine consumption; smoke; food purging, overeating, and avoidance contemplation; ratings of stress on a Visual Analog Scales (VAS)*;* and a verbal description of the previous hour. The second includes a momentary evaluation of ratings of stress [VAS and self-assessment manikins (Lang and Bradley, 1994)] and feelings toward food according to three dimensions: physical fullness, mental satisfaction, and intent to eat (Jiménez-Cruz et al., 2006; Forde, 2018). Each hourly report takes less than a minute to complete.

#### Stress Conditioning

**Figure 4:**
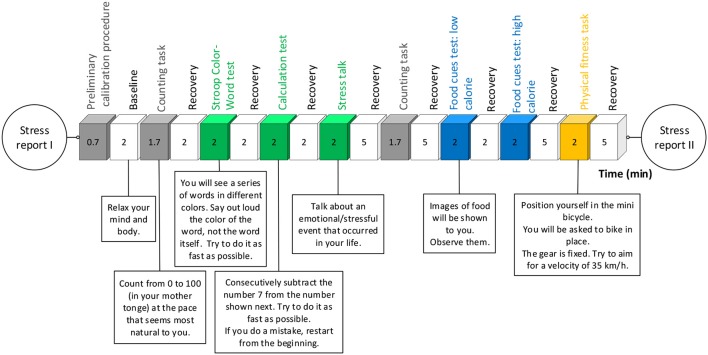
Stress task: timings, task modules, and task explanation.

We propose a customized stress task designed to elicit both mental and physical stress-related ANS activations. This multimodal task integrates mental arithmetic stimuli (Stroop Color-Word and calculation test), idiosyncratic stimulus (stress talk), ED specific stimulus (food cues test), physical stimulus (physical fitness task), baseline, and recovery rest phases (Figure 4). It takes ~60 min to complete. The modules and associated timings are indicated in Table 4 and a detailed description of each module is provided in Figure 4.

**Table 4:** Detailed description of the stress task modules and their purpose in our study.

**Task**	**Description**	**Purpose**
Counting task	Participant counts from 0 to 100 at a relaxed pace. This task takes place both at the beginning and at the end of the block involving speaking.	Control for the effect of speaking in the signals captured by the sensors. Not supposed to induce stress.
Stroop Color-Word test	Depicts how humans read faster than they identify and name colors. Participant is asked to name out loud, as fast as possible and under a limited time, the color of a word presented on the screen, while the word itself designates another color.	General mental activation. Social stressor. Previously, it was effectively used as a laboratory tool for stress induction Blondin and Renaud, 1997.
Calculation Test	Given a starting number displayed on the screen, the participant is asked to consecutively subtract the number seven from it, as fast as possible, while speaking out loud the result. In case there is a mistake, the participant has to restart the subtraction from the beginning.	General mental activation. Social stressor. Effectively used as a in clinical and healthy samples to induce psychophysiological activation Smets et al., 2016; Huysmans et al., 2018.
Stress Talk	Participant is invited to speak about one stressful or emotional event that occurred in her life. Researcher rates how stressful the content of the talk was on a VAS.	Idiosyncratic mental stimulus. Effectively used as a in clinical and healthy samples to induce psychophysiological activation Smets et al., 2016; Huysmans et al., 2018.
Food Cues Test	Visual cues, i.e., pictures, containing food items of low caloric and high caloric foods are consecutively presented on the screen, the participant is asked to observe them.	ED relevant mental stimulus.
Physical Resilience test	Consists on cycling in place on a mini-bicycle while trying to achieve and maintain the velocity of 35 km/h.	Mild physical task is performed to test the physiological response to physical activation.

The task is conducted in a dedicated room and monitored by a trained researcher. The task is displayed on a computer screen, each phase is temporized, and the progression is automatic. The screen is recorded (no audiovisual involving the participant is recorded) to appropriately annotate the sensor data according to the successive activities. Instructions on the activities related to each task module are provided on screen and vocalized by the researcher. The start of the experiment consists of a calibration procedure for subsequent alignment of signals across sensors. It consists of five sets of downward/upward torso movements, conducted while seating, and keeping both hands on top of the sensor on the chest. Relaxing sounds are reproduced during baseline and recovery phases, to calm the participant before and after each stimulus. The researcher keeps a neutral posture, and is allowed only standard interactions during the stimulus, such as *You have to speak faster*; *Please, continue*; *Don't give up*; *You can continue*. Performances are not evaluated during the task. At the beginning and end of the task, participants rate their stress levels and feelings toward food on a short self-report that includes questions 12–18 of the diary (Figure 3). The block of activities involving speaking (Counting task, Stroop Color-Word test, Calculation test, Stress talk) and the block of ED relevant stimulus (Food Cues Test) are switched for half the participants, to control for effects on bio-signals related to habituation to the study environment.

#### Debriefing and Compensation

At the end of the study day the participants must return the wearable devices and the diary. The participant is debriefed and if the diary contains at least eight reports, the participant receives a 10€ voucher for online shopping, as an appreciation for the participation.

#### Power Analysis

Power analysis estimation was performed to back up the study decisions in what comes to the size of the study sample. Toward this end we considered some simplifications and assumptions:

- We focus only on processing autonomic signals (no psychological variables).
- All autonomic signals will be analyzed toward extracting parameters to describe the ANS function (e.g., mean HR at baseline).
- Each participant will be treated as an observation, represented by a set of parameters (e.g., mean rest HR, mean HR during task, mean temperature from 10 h 30–13 h 30, mean SC at lunch time…).
- We assume a Gaussian distribution for all parameters.
- When comparing clinical and control groups only matched participants will be considered, hence in this situation both groups will have the same number of individuals.
- Gender, BMI, and Age are controlled by design, and activities during the stress task are typified.
- The clinical group has four ED subgroups and the control group has no subgroups.
- Both clinical and control group include a varied set of individuals, we assume these groups to present comparable parameter dispersion. When considering clinical subgroups, the dispersion of parameter values per group should be lower (i.e., individuals are more alike) except for EDNOS, which should have similar dispersion as the overall clinical group.

We drafted four analysis methodologies in accordance to the research questions proposed in the introduction section, and hereafter, we present the respective power calculations:

- Hypothesis 1: *During the stress task, the autonomic parameters from the clinical group differ from those of the control group*. Toward evaluating the statistical significance of each parameter to differentiate across groups (i.e., unpaired samples) a *t*-test for two samples is considered. The nominal variable is being a patient (true or false), and the measurement variable is one of the autonomic parameters (e.g., mean HR at baseline). We target high magnitude differences across groups [high effect size^2^, *d* = 0.8 (Cohen, 1988)], using a strict significance level^3^ (*p* = 0.01), with high power^4^ (*P* = 0.85). In this situation, using R toolboxes for the calculation (*R.pwr* library), the minimum sample size attained is 43 individuals per group.

- Hypothesis 2: *During an ambulant condition, the autonomic parameters from the clinical group differ from those of the control group*. We use the same formulation as in the previous hypothesis, assuming a *t*-test for two samples. Since data collected in uncontrolled conditions is prone to artifacts, this may affect the signal physiological content and increase the data variance. Therefore, we expect to find less sharp differences, hence we set a lower effect size in this case. We target medium magnitude differences across groups [medium effect size, *d* = 0.7 (Cohen, 1988)], using a strict significance level (*p* = 0.01), with a high power (*P* = 0.85). In this situation, we require at least 55 individuals per group (according to *R.pwr* library).
- Hypothesis 3: *Patient groups (AN, BN, BED, EDNOS) present different autonomic parameters*. We expect to find differences across patient groups based on autonomic signals throughout the full collection. If we assume the symptomatologic classification to have a parallel on physiology, then we expect to be able to find four distinct groups in the data, i.e., AN, BN, BED, and EDNOS. In this context we can use a one-way ANOVA test to verify the statistical significance of differences across groups. Targeting high magnitude differences [high effect size^5^, *f* = 0.4 (Cohen, 1988)], using a strict significance level (*p* = 0.01), with a high power (*P* = 0.85), we require at least 29 subjects per group (according to *R.pwr* library).
- As for the fourth research question, related to data driven clustering and classification of ED patients, both unsupervised and supervised machine learning techniques will be used and compared. The diagnostic classes (AN, BN, BED, EDNOS) will be considered as labels for the supervised methods. Power calculation for this analysis was omitted given its exploratory character.

In summary, to attain statistically relevant findings from this study we require at least 55 ED participants and respective controls to test hypothesis 1 and 2, and 116 ED participants to test hypothesis 3. The number of possible participants is highly variable, but we envision that in average two new clinical participants will be available on a weekly basis, and one new healthy match will be found. Considering that the time limit for the study is 60 weeks (15 months) related to project constraints, we will be able to include 120 ED participants and 60 controls. This scenario agrees with the numbers from the power analysis.

### Data Management

The data acquired in this study is stored and managed using Research Electronic Data Capture (REDCap) (Harris et al., 2009), a secure web-based tool to support research studies hosted at the University of Leuven. Data input and verification are performed by two different researchers to ensure data integrity. No direct identifiers such as name, phone number, or e-mails are kept in the REDCap database, and the data is pseudo anonymized according to a customized procedure before further processing. Local copies of the data are kept in encrypted hard drives and backed-up once a week.

### Data Analysis

**Figure 5:**
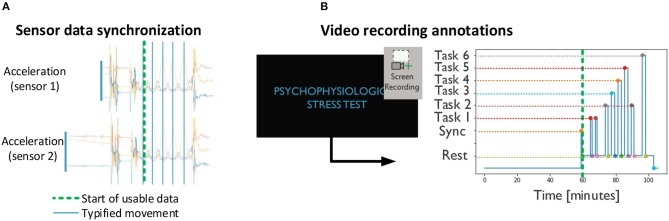
Preliminary procedures: **(A)** synchronization across devices based on acceleration and **(B)** annotation of stress task events based on screen recordings. **(A,B)** are combined according to the sync point (green dashed line).

A preliminary procedure for data analysis includes the generation of a set of annotations to contextualize the sensor data (Figure 5). Namely, the movement pattern resulting from the calibration procedure (Figure 4) is detected by visual inspection on the acceleration data of each sensor, and used to align the data across devices (Figure 5A). The task phases are identified by comparing individual frames of the screen recording to image templates. The starting point for relevant data is set to the moment at which the calibration procedure takes place (Figure 5, bold green line). Sensor data coherent with the stress task are then annotated based on the timings provided by the screen recording.

Quality of the data, in particular for the ambulant recording, is evaluated both via visual inspection and via automatic methods (Jones and Lederman, 2006; Boucsein et al., 2012; Kocielnik et al., 2013; Orphanidou et al., 2014; Smets et al., 2018).

Tonic and phasic components of the ANS activation are accounted *via* calculation of appropriate physiological parameters (e.g., HR and HRV (Task Force of The European Society of Cardiology and The North American, 1996; Acharya et al., 2006; Castaldo et al., 2015), SC level and responses (Boucsein, 2012) and skin temperature mean and variance) from the signals. For a complete list of the parameters that will be used as a starting point to our analysis (cf. section Power analysis) consult Table S5 on the Supplementary Materials of the article by Smets et al. (2018).

The laboratory data will be analyzed separately toward generating a numerical rating (Guo et al., 2018) of the physical fitness of the participant, that will be used as a covariate in the ambulant data analysis. In addition to age, BMI, comorbidities, symptoms, ED subtypes, and medications (highlighted in section Introduction), also time since admission, fitness, handedness, and actimetry (based on the accelerometer) will be controlled for.

## Discussion

While ED are mainly studied according to their psychological dimension, we proposed a new approach in which we intend to explore ED relation to ANS dysfunctions. To this end we designed a comprehensive protocol and successfully implemented it in a study in which we include physiological parameters from ECG, SC, and skin temperature. Participants are studied during the period of 1 day, with the expectation that longitudinal data can provide further insights than cross-sectional data. Recordings take place both in a lab session that calibrate individual physiological responses to different stressors, as well as in ambulatory, allowing participants to do the activities of their choice while the electrophysiological measurements are stored. At the time this article was written, we had collected data on 37 clinical subjects and 16 controls. Ongoing work focus on the implementation of a complete processing pipeline for data analysis and on the quantitative validation of our hypothesis using the collected dataset.

### Design Limitations and Notes for Future Enhancement

While the study design presents advantages over a cross-sectional one, because it includes continuous recording over about 8 h, the collection time can be still considered limited. While 1 day of data is enough to test our hypothesis, we cannot exclude punctual factors, not representative of the day to day life of the individual. In future research, conversion of the paper diary into an app and synchronized sensors data streaming directly to a cloud infrastructure, will reduce experimental burdens both for the participants and for the researchers allowing also for longer data collection period and even for easier implementation in clinical practice.

In what comes to physiological sensing, new non-invasive wearables methods have been recently developed that can be used in future trials to complement or substitute the current devices, for example multi-sensor wrist devices^6^ for HR, SC, and temperature monitoring, EMG devices^7^ to account for muscle tension induced by stress, EEG headsets^8^,^9^ (Emotiv, 2019) already used in arousal studies and attention, eye tracking glasses for monitoring blinking and pupil dilatation^10^,^11^, and hormonal sensing (Stanford EDU, 2018).

With the stress task presented we tried to assess broad set of test situations, nonetheless other variables could have been studied depending on the need to answer specific questions, e.g., emotion perception could be assessed in response to pictures from the International Affective Picture System (Joos et al., 2009; Gorini et al., 2010).

### Relevance

In the clinical context the evolution assessment of psychiatric patients relies mainly on self-reports and clinical observation. Information gaps between sparse appointments–when there is no hospital admission—are often bridged based on the patient reports, a recollection exercise that can be affected by momentary altered mental states. In ED, BMI is routinely measured to verify if weight goals are achieved, while cardiovascular assessment and blood analysis may be requested when the physical state of the patient is dubious. In these circumstances, most information gathered about the patient is sparse and subjective, often collected in a non-standardized way. In this backdrop, there is a recognized need for methodological enhancements in the field of mental health management. Nevertheless, in the past decades no major developments have been registered.

Recently mental health management sprouted attention outside medical and human sciences. This followed the technological developments that led to miniaturization of wireless sensing platforms, enhanced remote communications, increased computational power, and their broad availability to end users. The resulting enhanced possibilities for data collection and processing became an enabling factor for continuous follow-up and multifactorial analysis—seemingly matching the challenges of mental health management. Currently, there is a crescent interest and openness of the psychiatric research community to technology aided mental health management. Nonetheless, the topic is still highly enfolded in speculation and ethical concerns, and solid outcomes need to arise before the clinical community is convinced to adopt new technologies as an integral part of their methods.

If our hypothesis holds, we can further the understanding of psychopathology across ED and help establishing a dimensional biologically oriented nosology. For design reasons our study focuses on the treatment phase, when the patient has already reached the healthcare system. Which is nonetheless, a valid phase to introduce aiding technologies, namely, to help determining the patient psychopathological state, and track it during follow-up, toward modulating the treatment at each step.

## Ethics Statement

This study was carried out in accordance with the recommendations of the Commissie Medische Ethiek, UZ Leuven and in accordance to the Belgian law of 7 of May 2004 with written informed consent from all subjects. All subjects gave written informed consent in accordance with the Declaration of Helsinki. The protocol was approved by the Commissie Medische Ethiek, UZ Leuven.

## Author Contributions

NS-C, EV, and GS: conceptualization and investigation. NS-C, EV, CV, and WD: project administration. EV and CV: supervision. NS-C, GS, EV, CV, and WD: writing original draft, review, and editing.

### Conflict of Interest Statement

The authors declare that the research was conducted in the absence of any commercial or financial relationships that could be construed as a potential conflict of interest.

## Footnotes

^1^This classification was recently updated on the DSM-5 (American Psychiatric Association , 2013), though as the methods described in this article were drawn during the transition period, the DSM-IV-TR (American Psychiatric Association, 2000) notation is still adopted.

^2^Effect size phenomena is described by Cohen's *d* = (mean of clinical sample-mean of control sample)/average standard deviation. Cohen's recommendations for the *t*-test effect size: small effect size, *d* = 0.2; medium effect size, *d* = 0.5; high effect size, *d* = 0.8.

^3^P(Type I error): probability of finding a difference that actually does not exist.

^4^Power = 1–P(Type II error): probability of finding a difference that actually exists.

^5^Cohen's recommendations for the ANOVA effect size: small effect size, *f* = 0.1; medium effect size, *f* = 0.25; high effect size, *f* = 0.4.

^6^IMEC, “Chill+”. Available online at: https://www.imec-int.com/en/chill (accessed April 20, 2019).

^7^Plux, “Plux wearables: Muscle BAN.” Available online at: https://www.biosignalsplux.com/en/muscleban (accessed May 05, 2019).

^8^IMEC, “EEG headset.” Available online at: https://www.imec-int.com/en/eeg. (accessed May 05, 2019).

^9^Emotiv, “EEG headset comparison chart.” Available online at: https://www.emotiv.com/comparison/# (accessed May 05, 2019).

^10^IMEC, “Eye tracking with EOG.” Available online at: https://www.imec-int.com/nl/eog (accessed May 05, 2019).

^11^Tobii, “Eye tracking for research.” Available online at: https://www.tobiipro.com/ (accessed May 05, 2019).
